# Supplementary material for: Characterization of large-scale genomic differences in the first complete human genome
Source: Genome Biol. 2023 Jul 4;24:157. doi: 10.1186/s13059-023-02995-w (PMC10320979; doi:10.1186/s13059-023-02995-w)
Supplement: Supplementary file 1 — Additional file 1: Fig. S1. The visualization of four discrepant regions bydotplot. Fig. S2. Comparison of the gene length of CR1 in long-read human genome assemblies. Fig. S3. Gene structure differences in the discrepant regions. Fig. S4. The discrepant region contains CFHR1 and CFHR3. Fig. S5. The number of gene difference in deletions (total N = 68) and insertions (total N = 87) between GRCh38 and T2T-CHM13. Fig. S6. The distribution of the median CN s.d. Fig. S7.The distribution of intersected disease-related CNVs. Fig. S8. The SGPD read-depth genotyping of KLRC2 in different human populations. Fig. S9. The haplotype structure of living human populations in T2T-CHM13. Fig. S10. SNV distribution in T2T-CHM13 and GRCh38 of two chromosome regions. Fig.S11. CN of KLRC2 and KLRC3 for NHP population level. Fig. S12. The syntenic relationship of KLRC gene cluster region between human and different apes. Fig. S13. The syntenic relationship of KLRC gene cluster region between human and monkey genomes. Fig. S14. The phylogenetic tree of KLRC2 and KLRC3 with mouse lemur as outgroup. Fig. S15. The pi diversity of the KLRC gene cluster based on the 94 long-read genome assemblies. Fig. S16. Selection pressure testing using aBSREL on KLRC3 clade. Fig. S17.Comparison of the genomic region of the inversion containing a gap in GRCh38. Fig. S18. The syntenic relationship of GSTM gene cluster region between human and NHPs. Fig. S19. The phylogenetic tree of GSTM in primates. Fig. S20. The CN of KLRC3 in different human populations. [file 13059_2023_2995_MOESM1_ESM.pdf]

Supplementary Figures

A refined characterization of large-scale genomic differences in the first complete human genome

Xiangyu Yang†, Xuankai Wang†, Yawen Zou, Shilong Zhang, Manying Xia, Lianting Fu, Mitchell R. Vollger, Nae-Chyun Chen, Dylan J. Taylor, William T. Harvey, Glennis A. Logsdon, Dan Meng, Junfeng Shi, Rajiv C. McCoy, Michael C. Schatz, Weidong Li, Evan E. Eichler, Qing Lu, Yafei Mao*

*Corresponding author: yafmao@sjtu.edu.cn

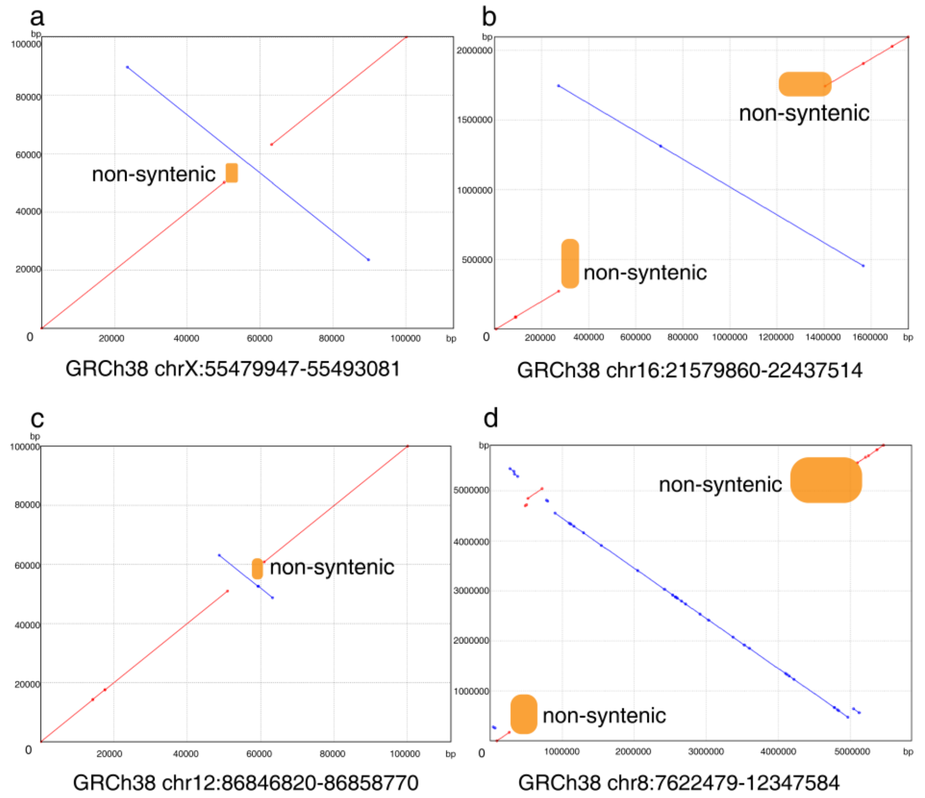

Fig. S1. The visualization of four discrepant regions by dotplot. The dotplots of the four different large-scale discrepant regions containing six 'non-syntenic' regions. The previous studies found the breakpoint of those inversions (orange area) rather than the whole inversion coordinate. The x-axis represents the GRCh38 coordinate and the y-axis represents the T2T-CHM13 coordinate. GRCh38 inversions shown for (a) chrX:55479947-55493081, (b) chr16:21579860-224437514, (c) chr12:86846820-86858770, and (d) chr8:7622479-12347584.

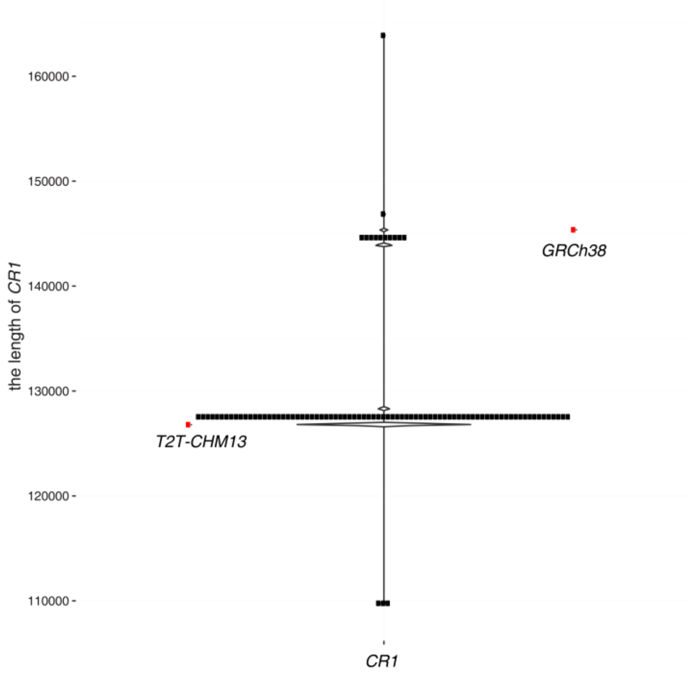

Fig. S2. Comparison of the gene length of *CR1* in long-read human genome assemblies. The violin plot shows the distribution of *CR1* length in the 94 long-read human genome assemblies. Red dots indicate *CR1* gene length in T2T-CHM13 and GRCh38. Black dots indicate the length of *CR1* in the 94 long-read human genome assemblies.

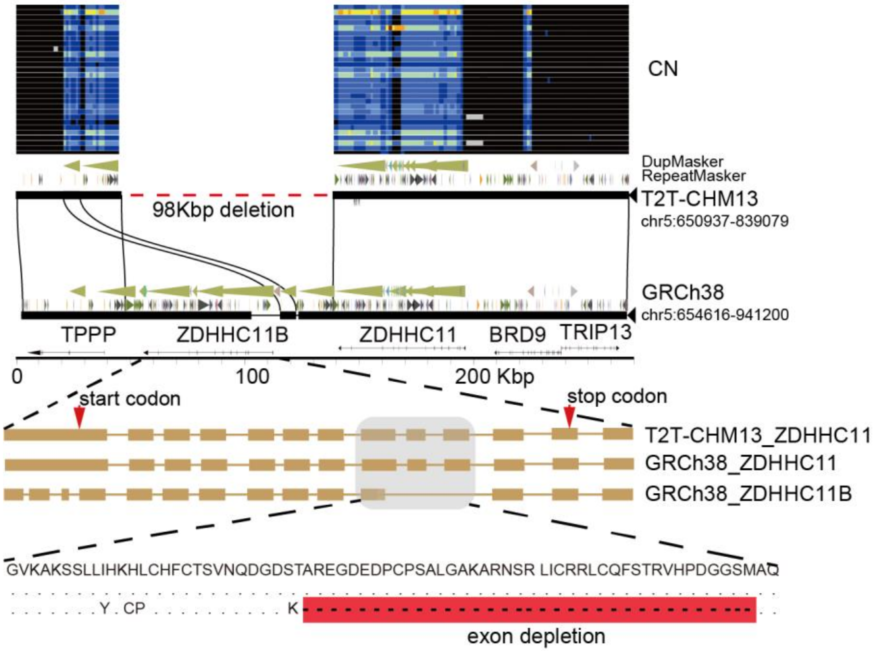

Fig. S3. Gene structure differences in the discrepant regions. The depletion of ZDHHC11B in the T2T-CHM13 genome assembly by a ~98 kbp deletion. The CN heatmap inferred from SGPD is shown in the top panel. The miropeat synteny relationship shows structural variation with repeat, duplication, and gene annotation. The exon schematic with amino acid alignment shows the gene model difference in the two assemblies.

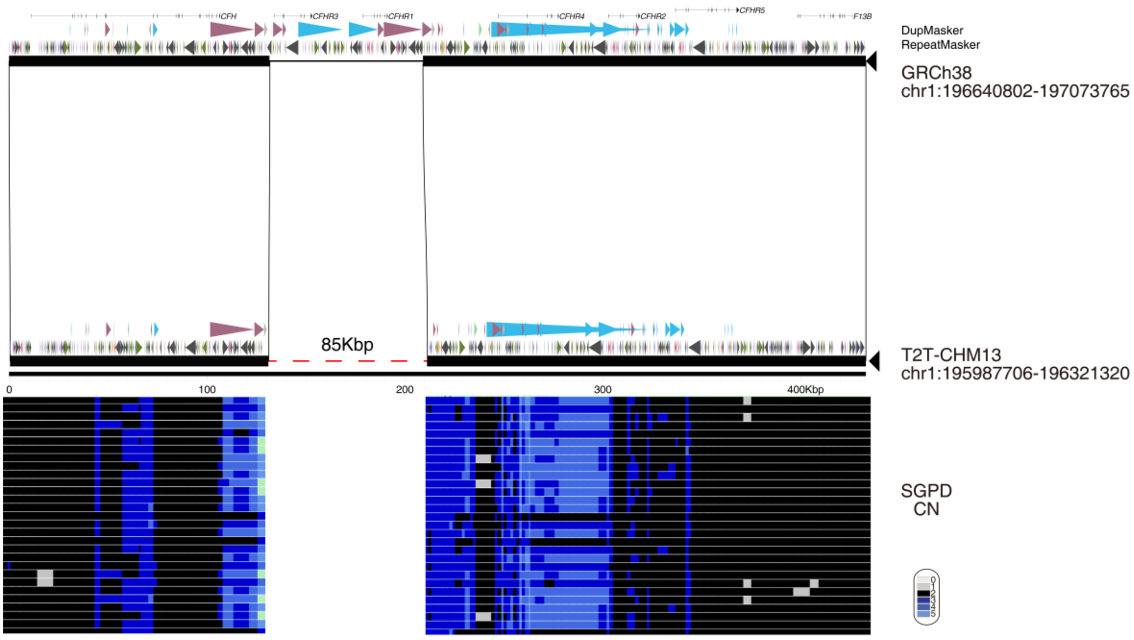

Fig. S4. The discrepant region contains *CFHR1* and *CFHR3*. The depletion of *CFHR1* and *CFHR3* by an 85 kbp deletion variant. The CN heatmap shows the CN of the discrepant region.

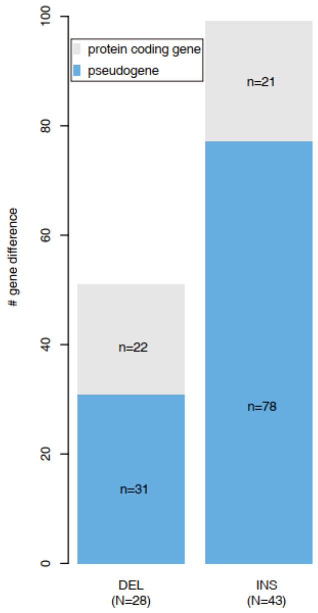

Fig. S5. The number of gene difference in deletions (total N = 68) and insertions (total N = 87) between GRCh38 and T2T-CHM13. Barplot shows the number of genes different (Y axis) in discrepant genomic regions between GRCh38 and T2T-CHM13. 28 deletion regions (N = 28, 28/68) and 43 insertion regions (N = 43, 43/87) include 53 genes (22 protein coding genes and 31 pseudogenes) and 99 genes (21 protein coding genes and 78 pseudogenes), respectively.

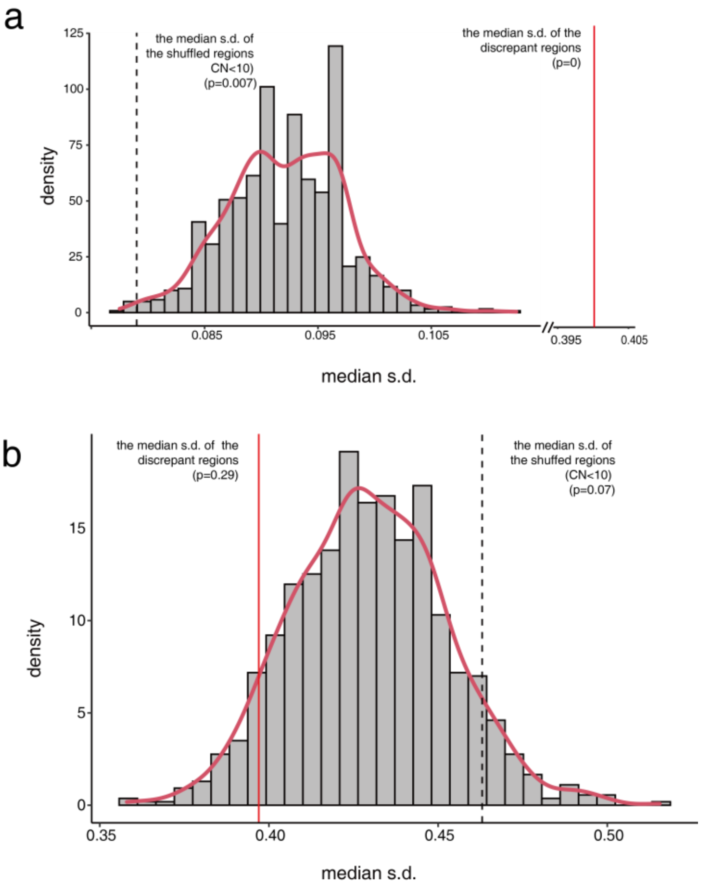

Fig. S6. The distribution of the median CN s.d. (a) Median s.d. of the 131 discrepant regions is significantly higher than the whole genome-wide simulation. (b) No significant difference between the median CN s.d. of the 131 discrepant regions (median=0.46) and that of the CN variable genomic regions (median=0.4, empirical p=0.07).

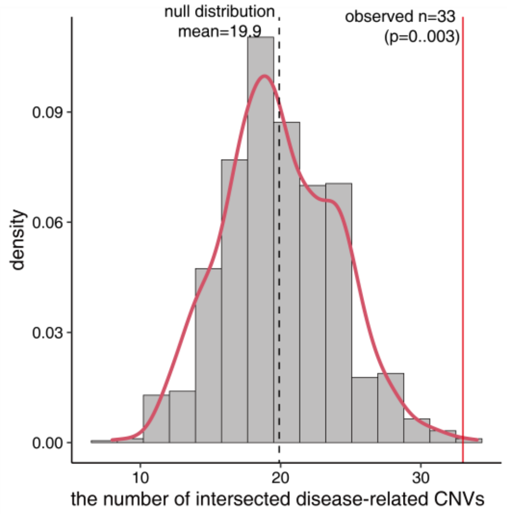

Fig. S7. The distribution of intersected disease-related CNVs. With genome-wide permutation analysis, we found some discrepant regions are more likely associated with the reported disease-related CNVs.

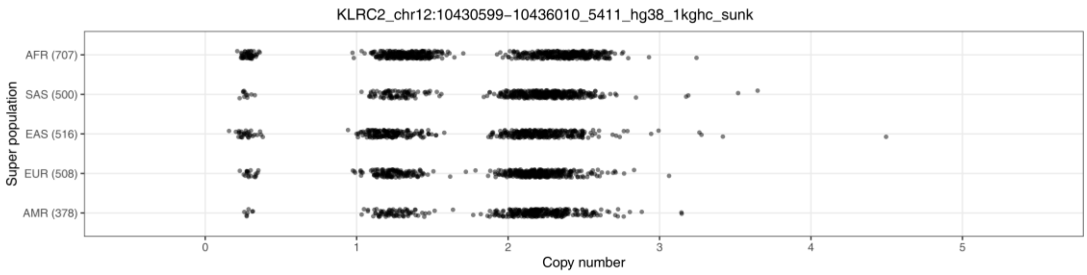

Fig. S8. The SGPD read-depth genotyping of *KLRC2* in different human populations. The CN of *KLRC2* in different populations present the CN polymorphism.

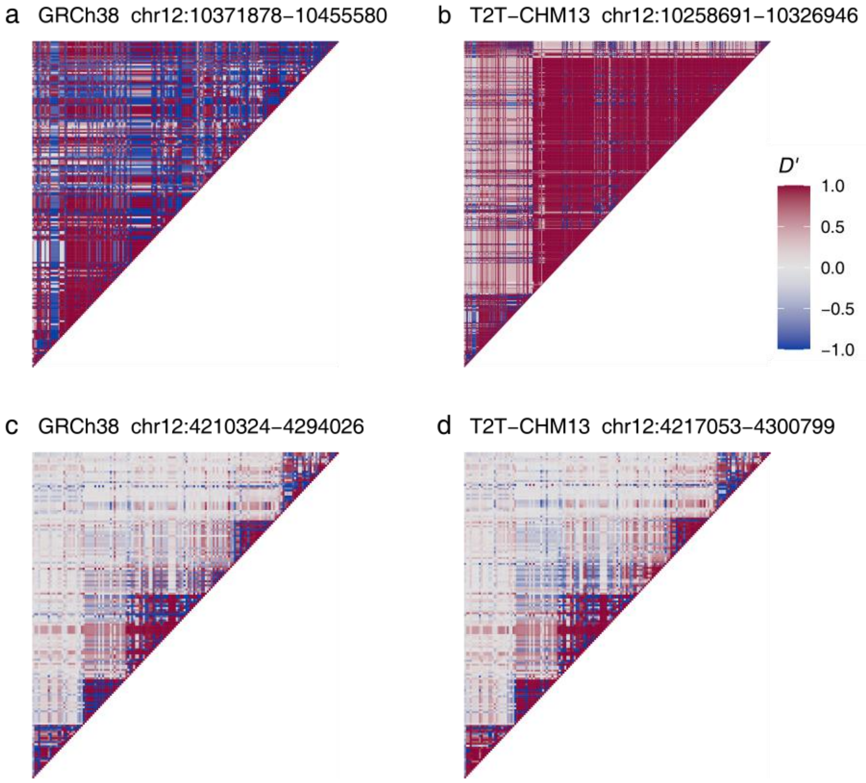

Fig. S9. The haplotype structure of living human populations in T2T-CHM13. LD at the *KLRC* locus extends much farther than the randomly selected control locus, which exhibits multiple, shorter haplotype blocks, potentially reflecting differences in the history of recombination within the regions.

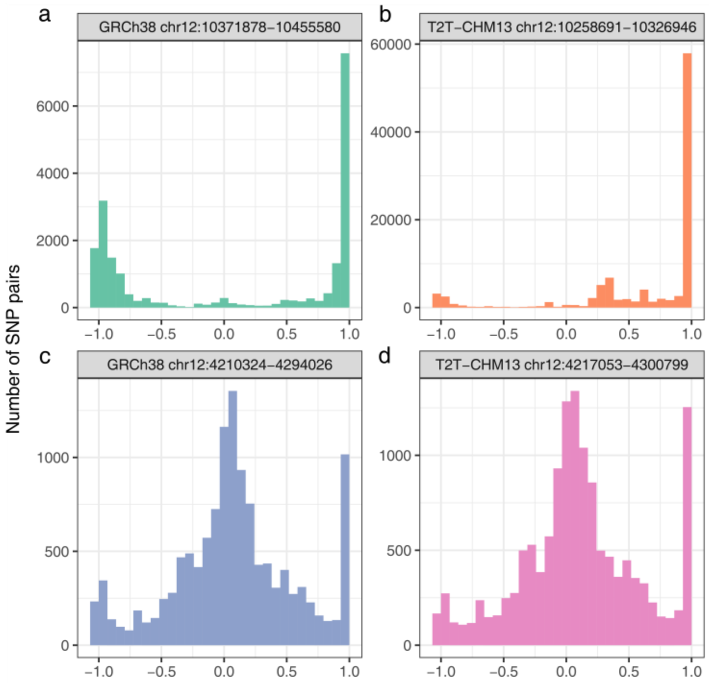

Fig. S10. SNV distribution in T2T-CHM13 and GRCh38 of two chromosome regions. Regions shown are (a) GRCh38 chr12:10371878-10455580, (b) T2T-CHM13 chr12:10258691-10326946, (c) GRCh38 chr12:4210324-4194026, and (d) T2T-CHM13 chr12:4217053-4300

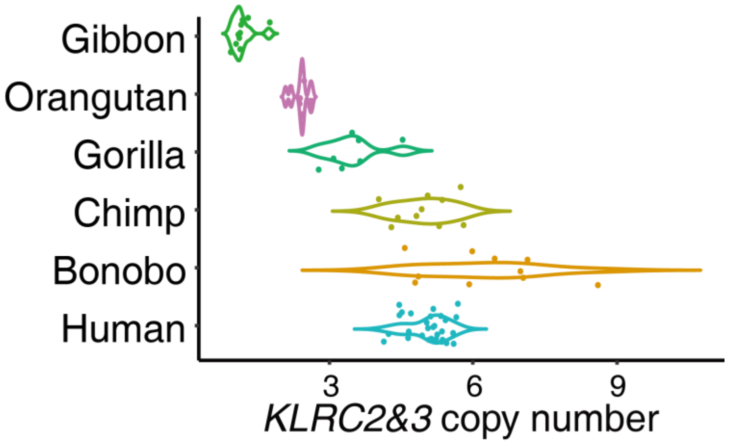

Fig. S11. CN of *KLRC2* and *KLRC3* for NHP population level. African great apes show the highest CN of *KLRC2* and *KLRC3* versus other primates.

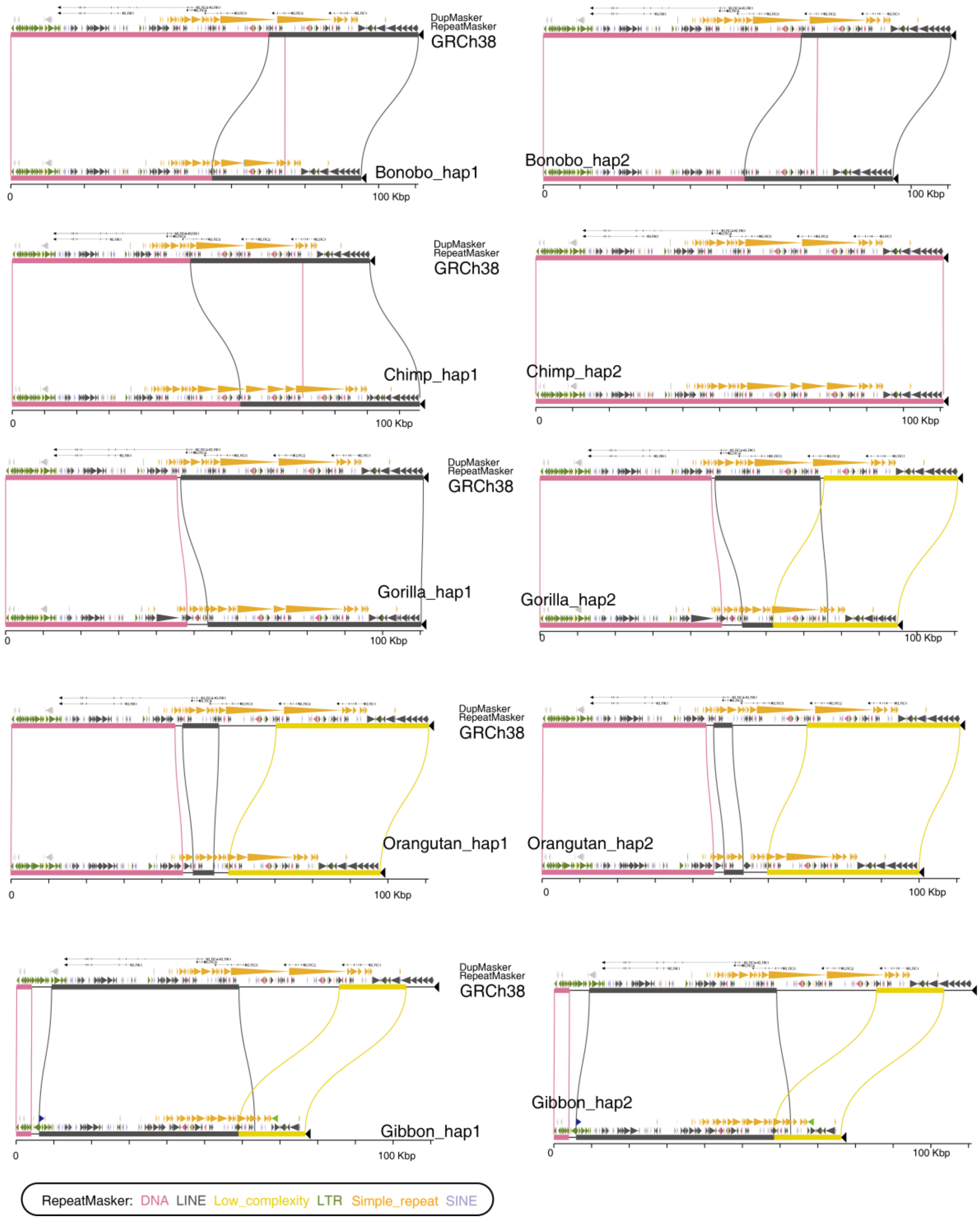

Fig. S12. The syntenic relationship of *KLRC* gene cluster region between human and different apes. *KLRC2* and *KLRC3* are deleted in two gibbon genome assemblies.

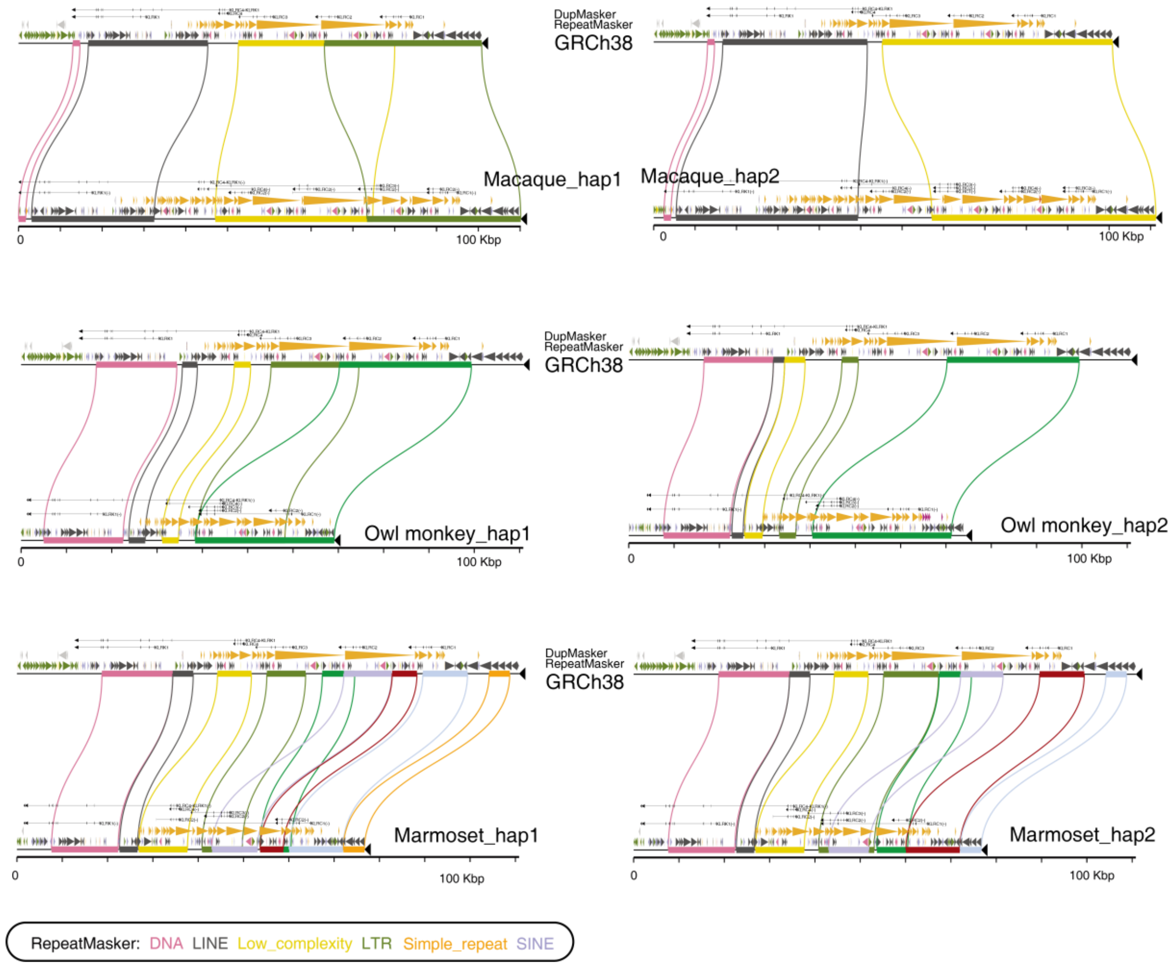

Fig. S13. The syntenic relationship of *KLRC* gene cluster region between human and monkey genomes. The duplication of *KLRC2* is found in macaques.

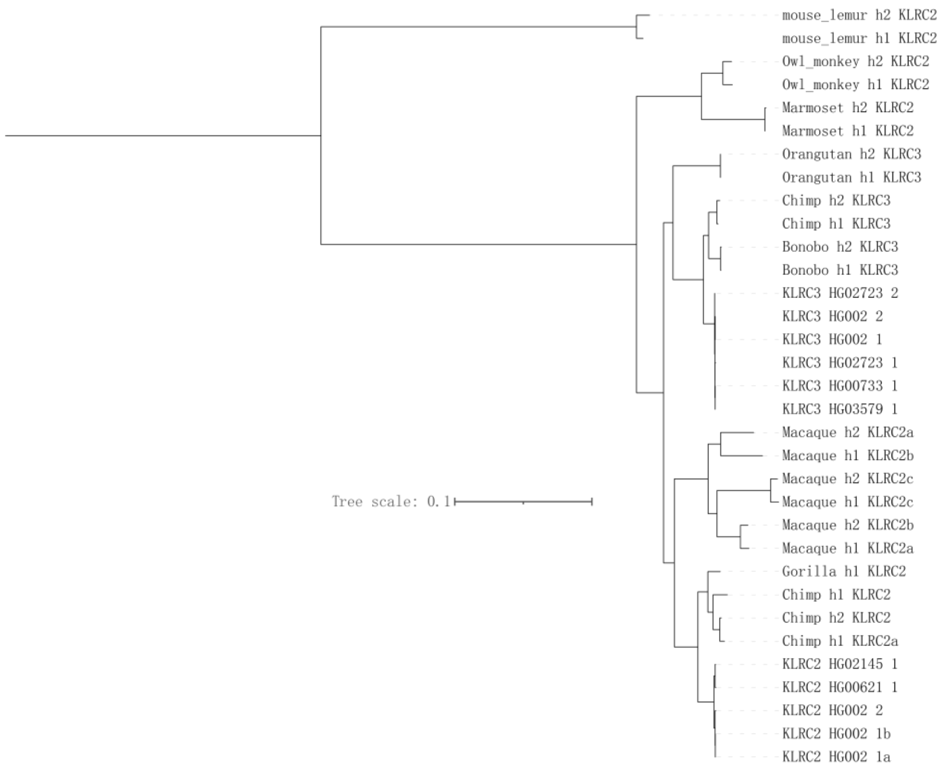

Fig. S14. The phylogenetic tree of *KLRC2* and *KLRC3* with mouse lemur as outgroup. The phylogenic tree of *KLRC2* and *KLRC3* with ~5.5 kbp genomic region suggests *KLRC2* and *KLRC3* are duplicated at the common ancestor of apes and Old-World monkey.

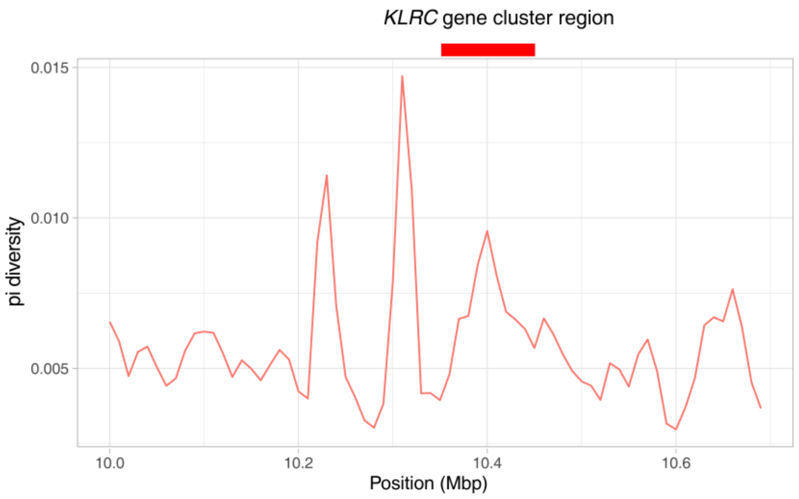

Fig. S15. The pi diversity of the *KLRC* gene cluster based on the 94 long-read genome assemblies. We did not obverse any significant pi diversity changes in the region. The red box represents the *KLRC* gene cluster region.

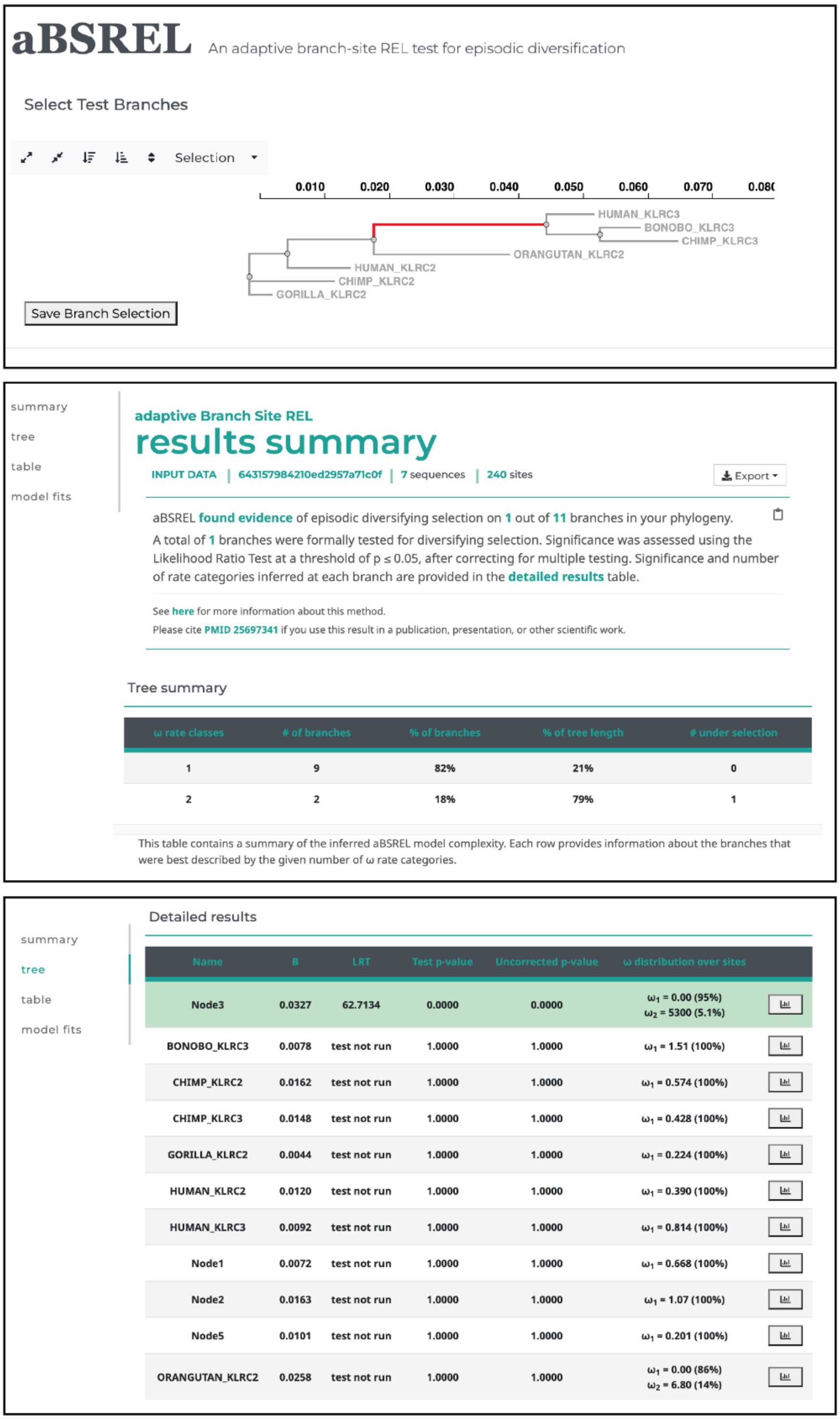

Fig. S16. Selection pressure testing using aBSREL on *KLRC3* clade. The aBSREL model suggested that node 2 is under selection with a *p*-value of $\leqslant 0.05$.

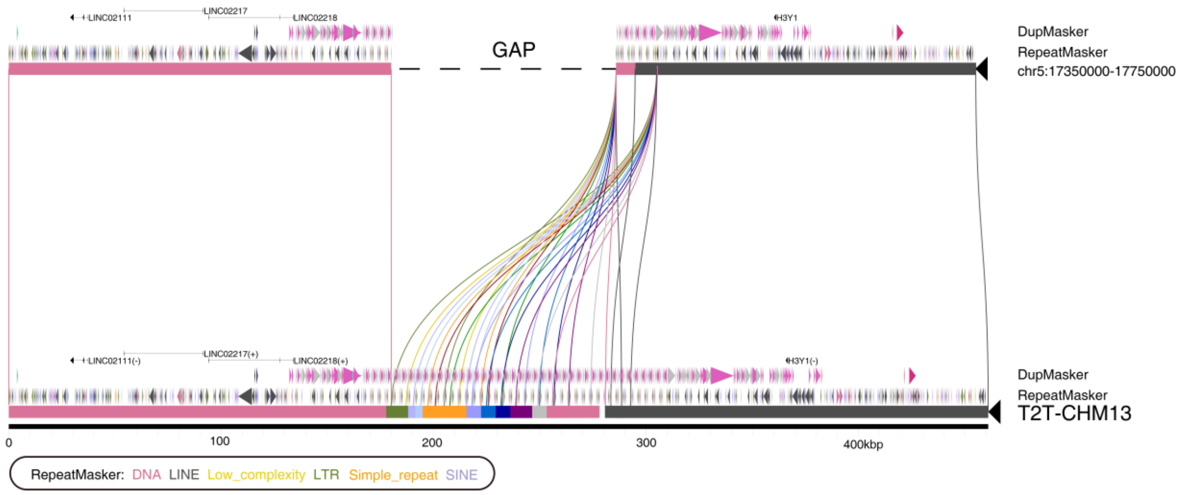

Fig. S17. Comparison of the genomic region of the inversion containing a gap in GRCh38. The miropeat synteny relationship of the region shows an inversion between GRCh38 and T2T-CHM13. There is a gap in GRCh38 resulting in a false negative call in the previous study.

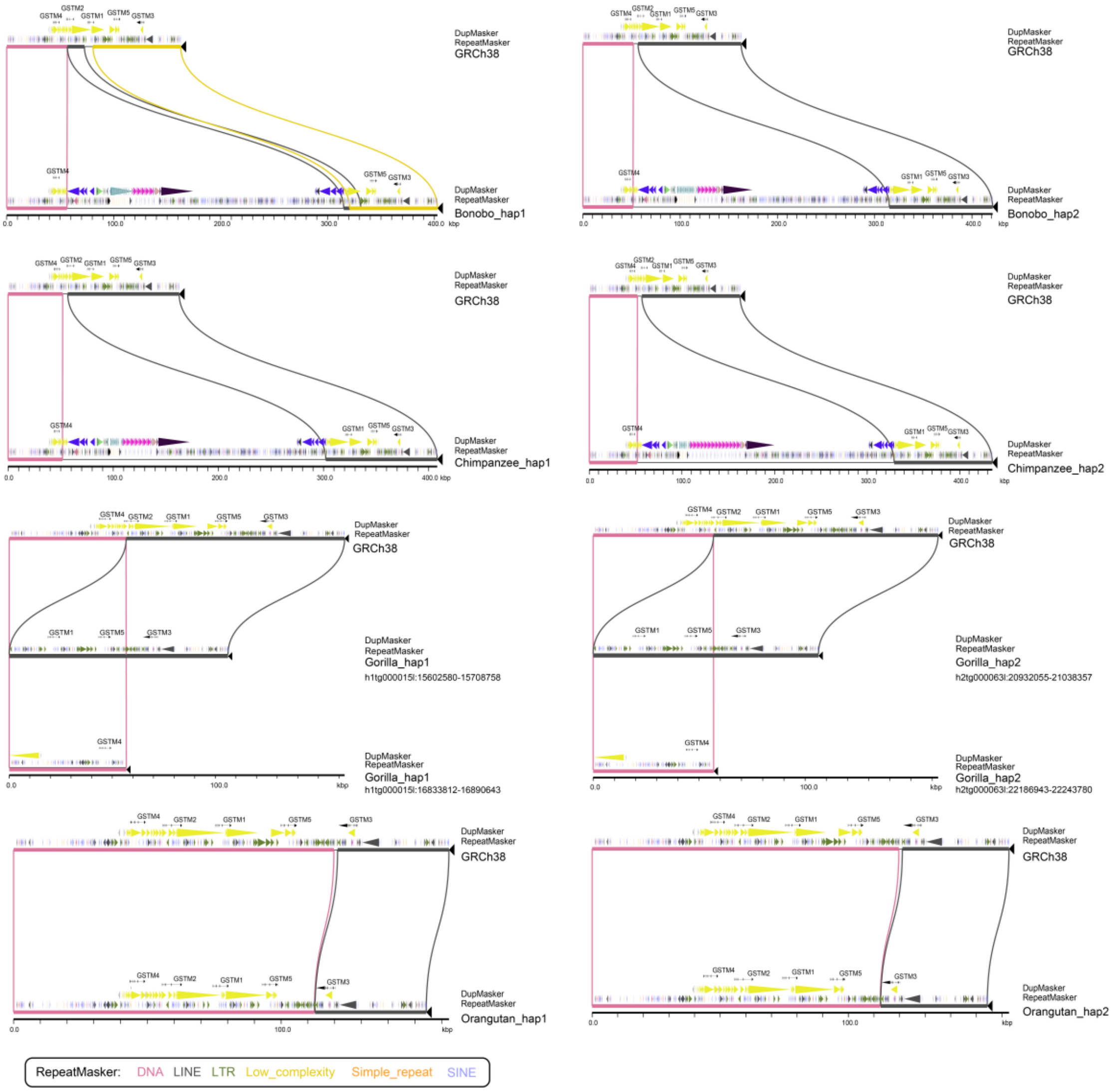

Fig. S18. The syntenic relationship of *GSTM* gene cluster region between human and NHPs. The copy number *GSTM* is variable in primates and *GSTM2* is deleted in chimpanzee, bonobo, and gorilla genome assemblies.

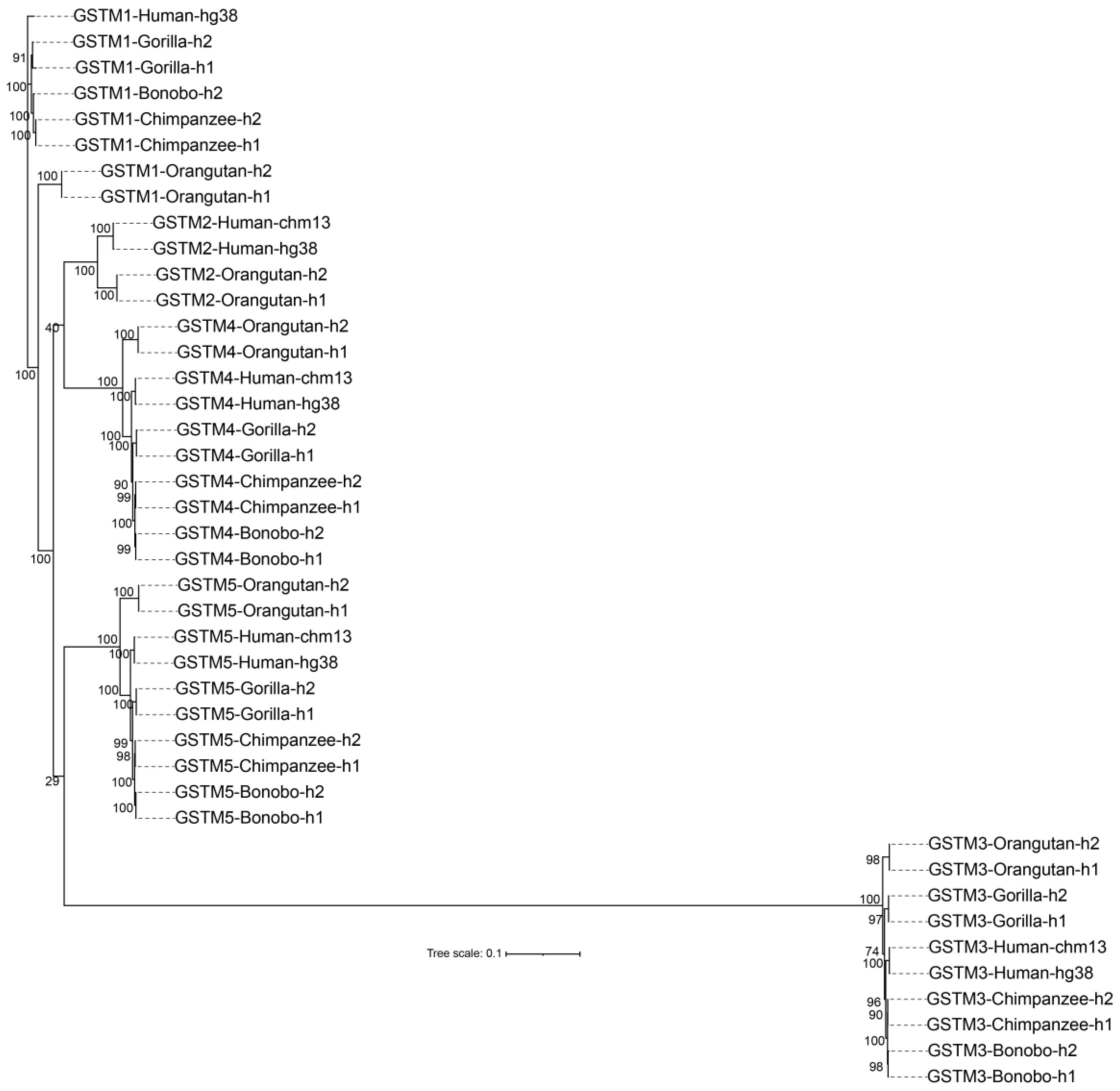

Fig. S19. The phylogenetic tree of *GSTM* in primates. The phylogenetic tree of *GSTM* reconstructed with 4.1 kbp genomic regions suggests that *GSTM1* and *GSTM2* gene recurrently mutated during primate evolution.

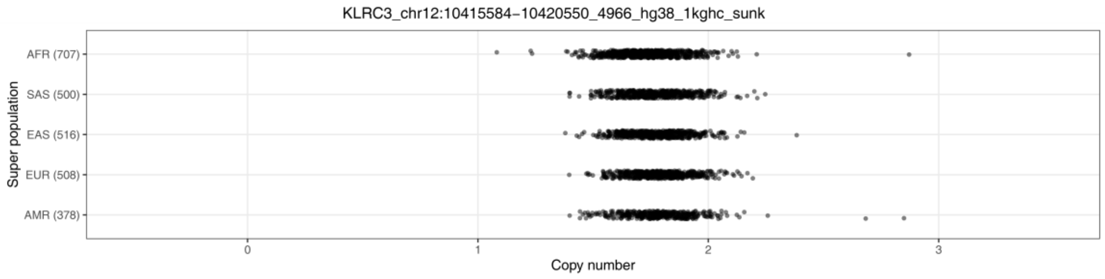

Fig. S20. The CN of *KLRC3* in different human populations. The result of the SGPD read depth genotyping suggests there is no CN variation of *KLRC3* in humans.
